# Glymphatic Dysfunction: A Bridge Between Sleep Disturbance and Mood Disorders

**DOI:** 10.3389/fpsyt.2021.658340

**Published:** 2021-05-07

**Authors:** Tao Yan, Yuefeng Qiu, Xinfeng Yu, Linglin Yang

**Affiliations:** ^1^Department of Psychiatry, Changxing People's Hospital, Huzhou, China; ^2^Department of Psychiatry, Zhejiang Hospital, Hangzhou, China; ^3^Department of Radiology, The Second Affiliated Hospital, Zhejiang University School of Medicine, Hangzhou, China; ^4^Department of Psychiatry, The Second Affiliated Hospital, Zhejiang University School of Medicine, Hangzhou, China

**Keywords:** glymphatic system, depression, sleep, bipolar disorder, astrocyte, aquaporin-4

## Abstract

Mounting evidence demonstrates a close relationship between sleep disturbance and mood disorders, including major depression disorder (MDD) and bipolar disorder (BD). According to the classical two-process model of sleep regulation, circadian rhythms driven by the light–dark cycle, and sleep homeostasis modulated by the sleep–wake cycle are disrupted in mood disorders. However, the exact mechanism of interaction between sleep and mood disorders remains unclear. Recent discovery of the glymphatic system and its dynamic fluctuation with sleep provide a plausible explanation. The diurnal variation of the glymphatic circulation is dependent on the astrocytic activity and polarization of water channel protein aquaporin-4 (AQP4). Both animal and human studies have reported suppressed glymphatic transport, abnormal astrocytes, and depolarized AQP4 in mood disorders. In this study, the “glymphatic dysfunction” hypothesis which suggests that the dysfunctional glymphatic pathway serves as a bridge between sleep disturbance and mood disorders is proposed.

## Introduction

Mood disorders are a group of complex debilitating psychiatric diseases identified by symptoms centered on markedly disrupted emotions, including major depressive disorder (MDD) and bipolar disorder (BD) (1). Due to their high prevalence, the risk for recurrence and suicide, they remain a serious health concern worldwide (2, 3). However, the exact neurobiological mechanisms underlying mood disorders remain unclear, resulting in unsatisfactory treatment (2, 3).

Sleep disturbance is a common concomitant and prodromal symptom of mood disorders (1, 4, 5). Specifically, both the two processes of sleep regulation—circadian oscillator and sleep pressure—are disrupted in mood disorders (4, 6). On one hand, circadian rhythms are approximately 24-h patterns in physiology and behavior, which are regulated by molecular clocks in the suprachiasmatic nuclei (SCN) of the hypothalamus (7). Mounting evidence suggests that there are abnormalities of the clock genes in mood disorders, such as single nucleotide polymorphisms (SNPs) (8–13), gene expression (14, 15), and gene–gene interactions (8). Excitingly, antidepressants including fluoxetine (16–18), ketamine (19, 20), and agomelatine (21) can reset the circadian clock along with the amelioration of mood symptoms. On the other hand, sleep pressure fluctuates with the sleep–wake cycle (6). Whereas, disturbance of the sleep–wake cycle has often been reported in mood disorders (22–24). Disturbed sleep architecture, especially decreased percentage of stage 3 non-rapid eye movement sleep (NREM III), represents decreased homeostatic drive for sleep (6). Actually, NREM III serves as a deep and recovery sleep, playing a vital role in the operation of the glymphatic system, and clearance of metabolic wastes (25, 26).

The glymphatic system is considered as an effective waste-removal system in the brain, which facilitates the exchange between the cerebrospinal fluid (CSF) and interstitial fluid (ISF), along with the potentially neurotoxic proteins such as amyloid-β (Aβ) (27), tau protein (28), and α-synuclein (29). Therefore, glymphatic impairment caused by sleep disturbance results in protein aggregation and increased risk for neurological diseases, such as Alzheimer's disease (AD) (30), Parkinson's disease (PD) (31), stroke (32, 33), and idiopathic normal cranial pressure hydrocephalus (iNPH) (34, 35). The water channel protein aquaporin-4 (AQP-4) is highly expressed on astrocytic endfeet and exerts significant influence in glymphatic transport (36). At present, accumulating evidence suggests the presence of abnormal astrocytes (37–43), depolarized AQP-4 (44–46), and dysfunctional glymphatic system (47, 48) in mood disorders. Therefore, we speculated that glymphatic dysfunction serves as an imperative intermediary factor between sleep disturbance and mood disorders.

In this study, we integrated available data from both animal and human studies regarding sleep in mood disorders and highlighted the core role of the glymphatic system. Furthermore, we discussed the glymphatic system dysfunction in mood disorders and identified the potential therapeutic opportunities for mood disorders based on sleep regulation and the glymphatic pathway.

## Sleep Disturbance and Mood Disorders

### The Model of Sleep Regulation

**Figure 1:**
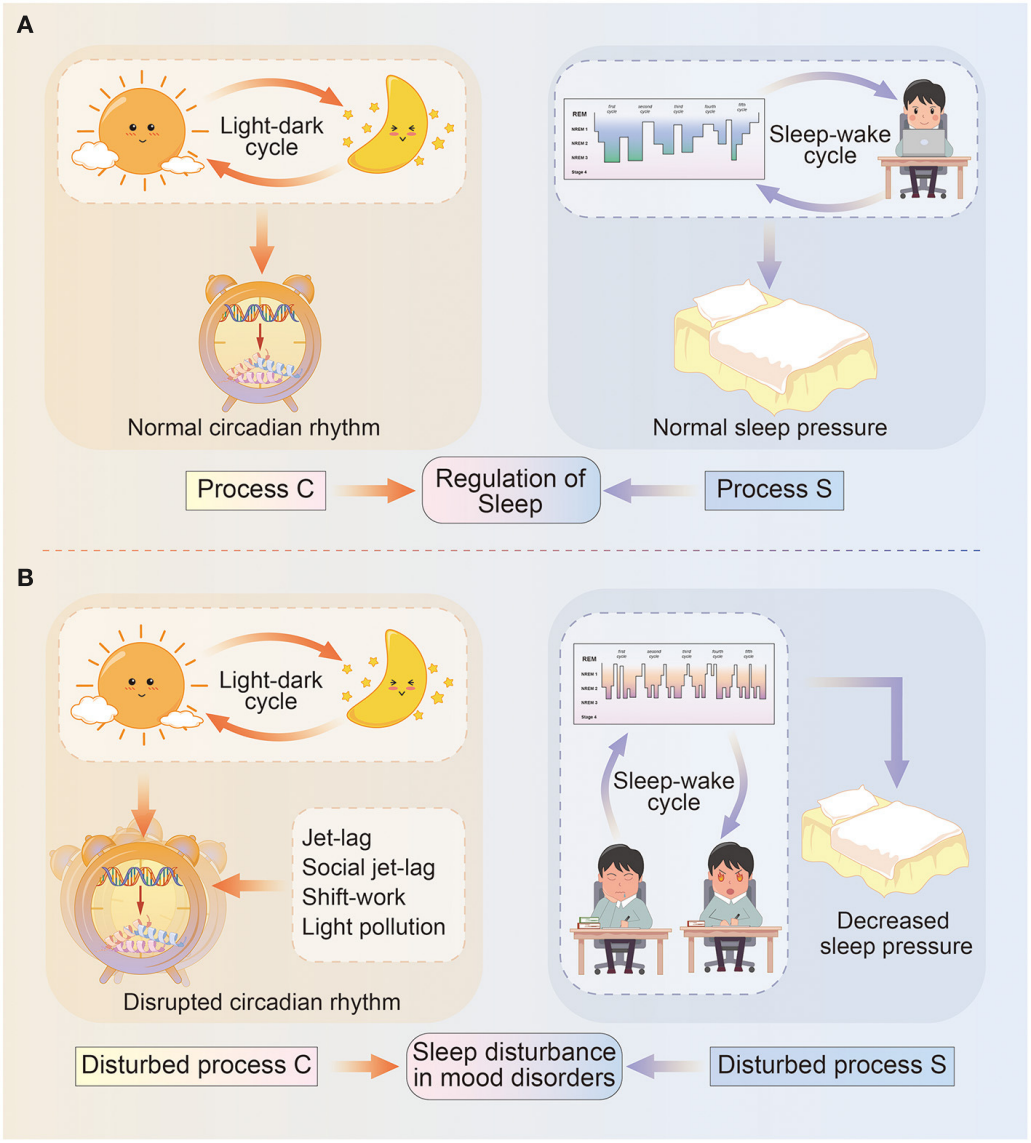
Diagram illustrating the two-process model of sleep regulation. **(A)** In normal circumstances, sleep regulation depends on the interaction between process C and process S. Specifically, process C represents the circadian rhythm driven by light–dark cycles, and circadian genes deliver circadian information via transcriptional–translational feedback loops and control physical and behavioral states. Process S means sleep pressure influenced by sleep–wake cycles, and include sleep architecture and daytime wakefulness. **(B)** In mood disorders, circadian rhythms (process C) are misaligned with light–dark cycles due to events such as jet-lag, social jet-lag, shift-work, light pollution, and so on; while sleep pressure (process S) is remarkably decreased due to longer sleep onset latency, a higher percentage of REM sleep, daytime sleepiness, or reduced need for sleep.

The classical two-process model of sleep regulation was first proposed by Borbély, and it consists of the process controlled by the circadian oscillator (Process C) and the homeostatic drive for the sleep–wake cycle (Process S). The two processes closely interact with each other but are also relatively independent (6) (Figure 1).

Circadian rhythms (Process C) are approximately 24-h rhythms in physiology and behavior, which are primarily driven by a hierarchy of cellular pacemakers located in the SCN (7). The most common measurements of the circadian rhythm are core body temperature and endogenous melatonin, other than the chronotype or morningness-eveningness (49). In fact, circadian rhythms are generated by a molecular clock in a network of positive and negative feedback loops. At the core of SCN timekeeping, the heterodimeric transcription factors CLOCK/BMAL1 translated from *CLOCK* and *Brain and muscle ARNT-like 1 (BMAL1)* genes, activate the *Period (PER1–3*) and *Cryptochrome* (*CRY1–2*) genes and initiate the circadian cycle. In turn, the dimer complex protein PER/CRY inhibit the activity of the CLOCK/BMAL1 proteins (50), exerting dominant effect in the negative feedback. As a critical complementary loop, the *BMAL1* transcription is activated by the retinoic acid-related orphan receptor (ROR) protein at night, and repressed by the nuclear receptors REV-ERB α/β (encoded by *NR1D1/2 genes*) at daytime (51), respectively. In addition, other clock genes also participate in the regulation of circadian rhythms. The *neuronal PAS domain protein 2 (NPAS2)* functions similarly to *CLOCK*, while *albumin gene D-site binding protein (DBP)* acts cooperatively with CLOCK/BMAL1 (52, 53). The *casein kinase I isoform* δ*/*ε *(CSNK1D/E)* regulates levels of PER by phosphorylation-mediated degradation, and thus inhibits the activity of CLOCK/BMAL1 (54). The *basic helix-loop-helix family 40/41* (*BHLHE40/41*, also known as *DEC1/2*) suppresses *PER* gene transcription via competing with *CLOCK-BMAL1* for *e-box* element binding (55). The *TIMELESS* gene is also conceived required for circadian rhythmicity, however, the exact role in human clockwork is still unclear (56). These circadian genes expression rise and fall in rhythm, contributing to the regulation of 24-h physical and behavioral cycles (15).

Process S, also referred to as the sleep pressure gradually accumulates during wakefulness and declines during sleep (6). Especially, as deep sleep (NREM III) dominates in the early phases of sleep and dwindles with decreasing sleep pressure in the late phases. Conversely, sleep deficit such as sleep deprivation results in a longer and deeper NREM III to achieve recovery (57), implying greater sleep pressure. Therefore, NREM III sleep is considered as a representation of sleep pressure (6). Sleep electroencephalogram (EEG) and actigraphy are effective assessments of sleep pressure to detect sleep architecture.

According to the two-process model, proper alignment of Process C and S is essential for recovery sleep. Otherwise, the daytime sleep fails to fulfill the homeostatic sleep drive, manifesting as lighter and lacking of recovery sleep (NREM III) (58). Moreover, the daytime sleep decreases sleep pressure, causing a negative influence on the more effective nighttime sleep.

### Sleep Disturbance in Mood Disorders

#### Disturbed Circadian Rhythms in Mood Disorders

Disruptions of the circadian rhythms are common in people exposed to jet-lag, social jet-lag, shift-work, as well as light pollution (light exposure at night) (59), and may lead to mood alterations (60, 61). Recently, a large population cross-sectional study (*n* = 91,105) using a wrist-worn accelerometer reported that lower relative amplitude of the circadian rhythm is associated with the lifetime prevalence of both MDD and BD (4). Individuals with circadian misalignment have higher depressive scores (62, 63). Moreover, a strong correlation between depressive symptoms and advances in dim light melatonin onset (DLMO) has been reported following an adjunctive multimodal chronobiological intervention organically combining psychoeducation, behavioral manipulation, and agomelatine intake (64). Bipolar disorder patients show delayed and decreased melatonin secretion during depressive and euthymic episodes (24, 65), with impaired psychosocial functioning and worse quality of life (24). In addition, manic and mixed episodes present with sustained phase advances, as well as a lower degree of rhythmicity corresponding to the severity of manic symptoms (66, 67). Apart from the daily (solar) cycle mentioned above, the lunar tidal cycles seem to entrain the mood cycles. In patients with rapid cycling BD, the periodicities in mood cycles have been observed to be synchronous with multiples of bi-weekly lunar tidal cycles (68).

The relationship between circadian rhythms and mood disorders is further supported by emerging genomic studies. In depressive cases, genetic association analyses have found SNPs in *PER2* (10870), *BMAL1* (rs2290035)*, NPAS2* (S471L), *CRY2* (rs10838524), *BHLHB2* (rs6442925), *CLOCK* (rs12504300), *CSNK1E* (rs135745), and *TIMELESS* (rs4630333 and rs1082214) (8, 9, 13). Single nucleotide polymorphisms in *CSNK1E* (rs135745), *TIMELES*S rs4630333, *CRY2* (rs10838524), *PER3* (rs707467 and rs10462020), *RORB* (rs1157358, rs7022435, rs3750420, and rs3903529), *REV-ERBA* (rs2314339) are strongly related to BD (8, 10–12, 69). In particular, *CLOCK* SNP rs1801260 contribute to the recurrence of mood episodes, while *CRY2* SNP rs10838524 is significantly associated to rapid cycling BD (10, 70). Moreover, the arrhythmic expression of circadian genes including *BMAL1, PER1–3, REV-ERBA, DBP*, and *BHLHE40/41*, has been observed in postmortem brain tissues of MDD patients (15). Reduced amplitude of rhythmic expression for BMAL1, REV-ERBα, and DBP has been reported in fibroblast cultures of 12 BD patients (14). Recently, Park et al. have explored gene–gene interactions of clock genes using the non-parametric model-free multifactor-dimensionality reduction (MDR) method, and revealed optimal SNP combination models for predicting mood disorders (8). Specifically, the four-locus model differs between MDD (*TIMELESS* rs4630333, *CSNK1E* rs135745, *BHLHB2* rs2137947, *CSNK1E* rs2075984) and BD (*TIMELESS* rs4630333, *CSNK1E* rs135745, *PER3* rs228669, *CLOCK* rs12649507), supporting the clinical observation of different circadian characteristics in two disorders.

#### The Unbalanced Homeostatic Drive of Sleep in Mood Disorders

The sleep–wake cycle is significantly affected by mood disorders. Firstly, a disturbed sleep–wake cycle is one of the most common diagnostic criteria for mood disorders. Individuals suffering from manic or hypomanic episodes often show a reduced demand for the sleep, while depressive patients experience insomnia or hypersomnia (1). Delayed sleep–wake phase and evening chronotype is common in patients with mood disorders (24, 71, 72), and strongly associated with the severity of mood symptoms (73). Sleep deficits predict a poor prognosis with a higher risk of suicide (74). Furthermore, both polysomnography and self-reported studies have revealed longer sleep onset latency, a higher percentage of rapid eye movement (REM) sleep, more fragmentation of the sleep/wake rhythm, and daytime dysfunction in patients with mood disorders during the remission state relative to healthy controls (22, 75, 76). More importantly, sleep disturbance often serves as a prodrome of manic or depressive episodes. Several retrospective studies have revealed that sleep disturbance is the most robust early symptom of manic episodes and the sixth most common prodromal symptom of manic episodes (5, 23). Recently, a 10-year prospective study among adolescents and young adults reported that the sleep problem is a risk factor for the development of BD (77). Sleep abnormalities have also been highly related to subsequent depression (23, 78, 79). Moreover, sleep deprivation is reported to trigger manic-like behavior in animal models (80). Thus, some researchers speculate that a disturbed sleep–wake cycle is probably a causal factor triggering mood episodes. However, because of ethical reasons, sleep generally cannot be manipulated in human research and this weakens the causal evidence between the sleep–wake rhythm and mood disorders.

#### Chronotherapeutic Treatments in Mood Disorders

In response to the vital roles that Process C and S play in the onset and course of mood disorders, chronotherapeutic interventions have been successfully used. Sleep deprivation combined with bright light therapy has been implicated in improving depressive symptoms (72, 81–83), while virtual darkness therapy via blue-light-blocking increases the regularity of sleep and a rapid decline in manic symptoms (84). These treatments exert great influence on mood recovery by resetting the circadian clock. Also, the hormone melatonin (MT) secreted by the pineal gland acts on the circadian clock via MT1 receptors (85, 86), while the MT agonist agomelatine shows important properties for phase shifts of the clock and anti-depressive effects (21). Additionally, agomelatine functions as an antagonist for 5-HT_2c_ receptors and modulates the master SCN clock via 5-HT innervations (87, 88). Similarly, other antidepressants can regulate the expression of the clock genes and thus affect the circadian rhythms (89). Fluoxetine, a selective serotonin reuptake inhibitor (SSRI) can shift electrical rhythms of the SCN and thus affect the behavior rhythm (16–18). Ketamine results in a rapid increase in glutamate level in the SCN and directly acts on NMDA receptors of the circadian clock in the epi-thalamic lateral habenula (LHb) (19, 20), suggesting that the rapid anti-depressive effects of ketamine might also be through the resetting of the circadian system (90). However, the mood stabilizer lithium is considered a clock-modifying drug in that it delays the sleep–wake cycle in healthy human and increase the length of the circadian period in non-human primates (91, 92). At the molecular level, lithium treatment can not only regulate the rhythm period via increasing *PER2* mRNA levels, but also significantly augment the oscillation amplitude PER2 and CRY1 protein rhythms via inhibiting the phosphorylation of glycogen synthase kinase 3β (GSK3B) (93, 94). Furthermore, the lithium efficacy is influenced by two *GSK3B* SNPs (rs334558 and rs3755557) (95). Considering all the above evidence, more pharmacological manipulations targeting the circadian rhythm and sleep drive are increasingly becoming plausible in the treatment of mood disorders.

Taken together, there seems to be a clear link between sleep disturbance and mood disorders, even though the underlying mechanisms remain unclear. The discovery of the glymphatic system provides researchers with insights into sleep-related diseases.

## Sleep and the Glymphatic System

### Overview of the Glymphatic System

The lymphatic system accounts for the clearance of ISF and it is also critical to both hydrostatic and homeostatic maintenance (96). With regard to lymphatic system in central nervous system (CNS), it consists of two interacting system, the glymphatic (glia-lymphatic) system and the meningeal lymphatic vessels (97). The glymphatic system is responsible for exchanging between CSF and ISF, and clearing solutes and metabolites from the brain parenchyma through a unique system of perivascular tunnels. More specifically, CSF produced by the choroid plexus and capillary influx is pumped deep into the brain parenchyma via arterial pulsation (36, 98). In the perivascular space (PVS), CSF exchanges with ISF, accompanied by clearance of soluble metabolic waste like Aβ (36). Indeed, large and eccentric PVS provides considerably less hydraulic resistance to CSF-ISF flow compared to concentric annular tunnel (99, 100). During the clearance of solutes, convection coexists with diffusion in the glymphatic system (101–103). It is argued that in the brain interstitium, small molecule transport is best explained by diffusion while convection becomes more predominant with increasing molecular size (104). However, the exact contributions of the two processes are highly dynamic and remain controversial, with one of the reasons being that the glymphatic influx and efflux are influenced by arousal state, pulse, respiration, body position, and more (98, 103, 105, 106). Moreover, CSF–ISF and solutes drain from the CNS via meningeal and cervical lymphatic vessels, as well as the cranial and spinal nerve roots (107, 108). Therefore, interference of the lymphatic system, such as ultraviolet photoablation of meningeal lymphatic vessels and ligation of cervical lymphatics, accounts for the stagnation of glymphatic flow and aggregation of metabolic wastes like Aβ (109, 110).

More importantly, the glymphatic system is supported by the water channel AQP4 which is primarily expressed by the astrocytic endfeet (36). Animals lacking AQP4 exhibit slower CSF influx and less interstitial solute clearance (70% reduction) (36, 111, 112). Deletion of the AQP4 in APP/PS1 transgenic mice results in increased interstitial Aβ plaque accumulation, cerebral amyloid angiopathy, as well as loss of synaptic protein and brain-derived neurotrophic factor in the hippocampus and cortex (113). However, it should be noted that the role of AQP4 in glymphatic clearance function are debated (103, 106). Smith et al. have found that *AQP4* gene deletion mice exhibited a similar Aβ distribution as wildtype mice, suggesting that *AQP4* gene deletion did not impair clearance of Aβ (114).

### Sleep-Dependent Glymphatic Cycling

Emerging evidence reveals that the function of the glymphatic system fluctuates daily along with the sleep–wake cycle. A two-photon imaging study reported a 60% increase in the interstitial space and two-fold faster clearance of Aβ in natural sleep or anesthesia mice compared with awake mice (27). A coherent pattern of slow-wave activity and CSF influx has been observed during NREM sleep in humans, supporting the exciting possibility of sleep-regulated glymphatic function (25). However, recent evidence using contrast-enhanced MRI has revealed that the glymphatic system is controlled by the circadian rhythm rather than by the sleep–wake cycle (115, 116). The parenchymal redistribution of contrast agent is lowest during the light phase and highest during the dark phase in fully awake rats, regardless of normal or reversed light–dark cycles (115). The diurnal variation of glymphatic cycling persists even under constant light or anesthesia, suggesting the hypothesis that endogenous circadian oscillations determine glymphatic function (116). The discrepancy may be related to the extreme differences in the circadian rhythm between humans and rodents (117). Rodents are nocturnal animals with opposite circadian phase, and they are also poly-phasic sleepers with relatively low sleep drive (118). Presently, the exact contributions of the light–dark cycle, sleep–wake cycle, and other physiological rhythms remain unknown (116). Further studies are warranted to confirm the circadian control of the glymphatic system in humans.

Surprisingly, the deletion of AQP4 effectively eliminates the circadian rhythm in glymphatic fluid transport (116). A recent genomic study reports that AQP4-haplotype influences sleep homeostasis in NREM sleep and response to prolonged wakefulness (119), providing supporting evidence for the sleep-dependent glymphatic pathway. The high polarization of AQP4 in astrocytic endfeet is under the control of the circadian rhythm, and thus, modulates bulk fluid movement, CSF–ISF exchange, and solutes clearance (116). Conversely, there is also evidence that astrocytes repress SCN neurons and regulate circadian timekeeping via glutamate signaling (120). Thus, astrocytes and AQP4 present a checkpoint for the functional glymphatic system during deep sleep.

Considerable evidence suggests a causal relationship between sleep and regulation of the glymphatic flow, thus modulating protein clearance. Sleep disturbance (including shorter total sleep time, sleep fragmentation, and lack of NREM III) causes suppressed glymphatic function and a decline in the clearance of metabolic waste, hence contributing to the development and progression of various neurological diseases including AD (30), PD (31), stroke (32, 33), and iNPH (34, 35).

Taken together, the glymphatic function is considered as a brain fluid transport with astrocyte-regulated mechanisms, while glymphatic dysfunction is intimately associated with neurological diseases, especially neurodegenerative diseases with cognitive decline (30, 31).

## Glymphatic Dysfunction in Mood Disorders

### Abnormalities of Glymphatic Flow, Astrocytes, and AQP4 in Depression

Individuals suffering from depressive episodes always show diverse cognitive decline (1), including attention, memory, response inhibition, decision speed, and so on. Depression has been considered as a prodrome of dementia (121), with increased Aβ deposition reported in an (18) F-florbetapir positron emission tomography (PET) imaging study (122). These observations raise the exciting possibility that wide-spread disruption of the glymphatic system exists in depression. Recent animal studies using chronic unpredictable mild stress (CUMS) model have provided supporting evidence for the glymphatic dysfunction in depression (47, 48) (Table 1). In the CUMS model, animals were exposed to the various stressors randomly for several weeks and injected with fluorescence tracers from cisterna magna to estimate the glymphatic function (47, 48). The CSF tracer penetration in the brain of CUMS-treated mice was significantly decreased, and recovered to the control level after fluoxetine administration or polyunsaturated fatty acid (PUFA) supplementation (47, 48). In parallel with the impaired glymphatic circulation, the increased deposition of Aβ has been observed (47, 48). Amyloid-β accumulation along the blood vessels, in turn, could impair glymphatic function by reducing PVS and increasing hydraulic resistance, and thus result in a more severe parenchymal build-up of Aβ and neuronal death (134). Another plausible explanation of PVS closure induced by CUMS is the alteration of arterial pulsation and compliance that triggered by neuroinflammation and restored by daily PUFA supplementation (48) (Table 1).

**Table 1:** Glymphatic flow, astrocytes, and AQP4 in animal studies.

**References**	**Studied cohort**	**Method**	**Main findings**
Xia et al. (47)	CUMS model mice	Injection of tracers, immunohistochemistry	Impaired glymphatic circulation and increased accumulation of Aβ42, which can be reversed by fluoxetine treatment. Downregulated AQP4 expression in cortex and hippocampus, which can be reversed by fluoxetine treatment.
Liu et al. (48)	CUMS model mice	Injection of tracers, immunohistochemistry	Impaired glymphatic circulation and cerebrovascular reactivity, which can be reversed by PUFA supplementation. Decreased Aβ40 clearance, which can be reversed by PUFA supplementation and escitalopram treatment. Decreased astrocytes and AQP4 expression, which can be reversed by PUFA supplementation and escitalopram treatment.
Gong et al. (123)	CMS model mice	Immunohistochemistry	Decreased hippocampal astrocyte is passed on to offsprings via an epigenetic mechanism.
Czéh et al. (124)	Chronic psychosocial stress mice	Immunohistochemistry	Fluoxetine treatment prevented the stress-induced numerical decrease of astrocytes.
Kinoshita et al. (125)	VNUT-knockout mice	Immunohistochemistry, qPCR	Fluoxetine increased ATP exocytosis and BDNF in astrocytes.
Hisaoka-Nakashima et al. (126)	Rat primary astrocytes, C6 astroglia cells	qPCR, ELISA, western blotting	Mirtazapine treatment increased mRNA expression of GDNF and BDNF in astrocytes.
Wang et al. (127)	Mice	Western blotting	Ketamine promotes the activation of astrocyte.
Lasič et al. (128)	Rat primary astrocytes	Structured illumination microscopy and image analysis	Ketamine induced cholesterol redistribution in the plasmalemma of astrocytes.
Xue et al. (129)	CUS model rats	Immunohistochemistry, qPCR	Repetitive TMS at 5 Hz increased the expression of DAGLα and CB1R in hippocampal astrocytes and neurons.
Taler et al. (130)	CUMS model rats	Immunohistochemistry, western blotting, ELISA	Lithium can attenuate the reduction of AQP4 and disruption of the neurovascular unit in hippocampus.
Wang et al. (131)	LPS-induced depression model mice	Immunohistochemistry, qPCR	Inhibition of activated astrocytes ameliorates LPS-induced depressive-like behavior.
Portal et al. (132)	Cx43 KD male mice	Immunohistochemistry, western blotting	Inactivation of astroglial connexin 43 potentiated the antidepressant-like effects of fluoxetine.
Kong et al. (133)	CMS model mice	Immunohistochemistry, western blotting	AQP4 knockout disrupted fluoxetine-induced enhancement of hippocampal neurogenesis, as well as behavioral improvement.

*Aβ, amyloid-β; AQP4, aquaporin-4; ATP, adenosine triphosphate; BDNF, brain-derived neurotrophic factor; CB1R, cannabinoid type 1 receptor; CMS, chronic mild stress; CUMS, chronic unpredictable mild stress; CUS, chronic unpredictable stress; Cx43 KD, connexin 43 knock-down; DAGLα, diacylglycerol lipase alpha; ELISA, enzyme-linked immunosorbent assays; GDNF, glial cell line-derived neurotrophic factor; GFAP, glial fibrillary acidic protein; LPS, lipopolysaccharide; mRNA, messenger RNA; PUFA, polyunsaturated fatty acid; qPCR, quantitative polymerase chain reaction; TMS, transcranial magnetic stimulation; VNUT, vesicular nucleotide transporter*.

During the neuroinflammatory response, reactive astrocytosis, and AQP4 depolarization have been widely reported in depression (48). Abundant evidence indicated astrocytic abnormalities in patients with depression (Table 2). Golgi-staining of postmortem tissues from depressed suicide cases has revealed reactive astrocytosis within the cingulate cortex (37). Additionally, glial fibrillary acidic protein (GFAP), one of the astrocyte-specific biomarkers, is reduced in depression-associated brain regions including the prefrontal cortex, cingulate cortex (38, 39), hippocampus (40), amygdala (41), locus coeruleus (44), cerebellum (146), thalamus, and caudate nuclei (42). A lower density of S100β-immunopositive astrocytes has been reported in the bilateral hippocampus and locus coeruleus of depressive patients compared to that of healthy controls (44, 135). Downregulated expression of AQP4 has been found in postmortem locus coeruleus and hippocampus in MDD patients (44, 136). More importantly, the reduction in astrocyte density is passed on to offsprings of depressive females via an epigenetic mechanism (123) (Table 1). Nevertheless, there are several contradictory results (Table 2). The density of astrocytes has been observed unchanged in the cingulate cortex and hippocampus of MDD patients (142, 144). A postmortem study using quantitative polymerase chain reaction (qPCR) have observed upregulated expression of GFAP and aldehyde dehydrogenase 1 family member L1 (ALDH1L1) in the basal ganglia of MDD patients (145). Another postmortem study using microarray analysis and qPCR has found upregulated expression of AQP4 in the prefrontal cortex of MDD patients. Obviously, the variety of studied methods involving Glogi-staining, Nissl-staining, qPCR, western blotting, and immunohistochemistry, contributes to the discrepancies.

**Table 2:** Astrocytes and AQP4 in patients with mood disorder.

**References**	**Studied cohort**	**Tested sample**	**Method**	**Main findings**
Torres-Platas et al. (37)	10 Depressed suicides, 10HC	Postmortem tissue	Golgi-staining	Reactive astrocytosis within the cingulate cortex of depressive patients.
Torres-Platas et al. (42)	22 Depressed suicides, 22HC	Postmortem tissue	Immunohistochemistry, qPCR	Downregulation of GFAP mRNA and protein in the mediodorsal thalamus and caudate nucleus of depressed suicides.
Webster et al. (38)	15MDD, 15BD, 15HC	Postmortem tissue	*In situ* hybridization	Decreased level of GFAP mRNA in the cingulate cortex of BD patients. Decreased level of GFAP mRNA in the cingulate cortex of MDD patients (not significantly).
Gittins et al. (39)	5MDD, 2BD, 9HC	Postmortem tissue	Immunohistochemistry	Decreased GFAP protein in the anterior cingulate cortex of patients with mood disorders.
Cobb et al. (40)	17MDD, 17HC	Postmortem tissue	Immunohistochemistry	Decreased GFAP-positive astrocytes in the left hippocampus of depressive patients.
Altshuler et al. (41)	11MDD, 10BD, 14HC	Postmortem tissue	Immunohistochemistry	Decreased GFAP-positive astrocytes in the amygdala of depressive patients. Unchanged GFAP-positive astrocytes in the amygdala of BD patients.
Bernard et al. (44)	12MDD, 6BD, 9HC	Postmortem tissue	*In situ* hybridization	Downregulated expression of GFAP, S100B and AQP4 in locus coeruleus of MDD patients.
Gos et al. (135)	9MDD, 6BD, 13HC	Postmortem tissue	Immunohistochemistry	Decreased S100β-immunopositive astrocytes in the bilateral hippocampus of depressive patients.
Medina et al. (136)	13MDD, 10HC	Postmortem tissue	Microarray analysis, qPCR	Downregulated AQP4 mRNA expression in hippocampus of MDD patients.
Feresten AH et al. (137)	34BD, 35HC	Postmortem tissue	Western blotting	Increased GFAP expression of in BA9 of BD patients. Unchanged levels of vimentin and ALDH1L1 in BA9 of BD patients.
Johnston-Wilson et al. (138)	19MDD, 23BD, 23HC	Postmortem tissue	Western blotting	Decreased GFAP-positive astrocytes in BA10 of BD patients.
Toro et al. (139)	15MDD, 15BD, 15HC	Postmortem tissue	Immunohistochemistry	Decreased GFAP-positive astrocytes in BA11/47 of BD patients.
Dean et al. (140)	8BD, 20HC	Postmortem tissue	Western blotting, qPCR	Increased S100β in BA40 of BD patients. Decreased S100β in BA9 of BD patients.
Hercher et al. (141)	20BD, 20HC	Postmortem tissue	Immunohistochemistry	Unchanged density of astrocytes in the frontal cortex of BD patients.
Williams et al. (142)	20MDD, 16BD, 20HC	Postmortem tissue	Immunohistochemistry	Unchanged density of astrocytes in the cingulate cortex of patients with mood disorder.
Pantazopoulos et al. (143)	11BD, 15HC	Postmortem tissue	Immunohistochemistry	Unchanged density of astrocytes in the amygdala and entorhinal cortex of BD patients.
Malchow et al. (144)	8MDD, 8BD, 10HC	Postmortem tissue	Nissl-staining	Unchanged density of astrocytes in the hippocampus of patients with mood disorder.
Barley et al. (145)	14MDD, 14BD, 15HC	Postmortem tissue	qPCR	Upregulated expression of GFAP and ALDH1L1 the basal ganglia of MDD patients. Upregulated expression of GFAP and ALDH1L1 the basal ganglia of BD patients (not significantly).
Fatemi et al. (146)	15MDD, 15BD, 15HC	Postmortem tissue	Western blotting	Decreased GFAP in the cerebellum of patients with mood disorders.
Steiner et al. (147)	9MDD, 5BD, 10HC	Postmortem tissue	Immunohistochemistry	No change in GFPA-immunopositive astrocytes of patients with mood disorder.
da Rosa et al. (148)	52 manic BD, 52HC	Serum	meta-analysis	Increased S100β levels in serum of patients with manic episodes.
Zhao et al. (46)	50BD II, 43HC	eDWI	ADCuh	Increased ADCuh values in bilateral SCP and cerebellar hemisphere, which positively associated with depressive scores.
Iwamoto et al. (149)	11MDD, 11BD, 15HC	Postmortem tissue	Microarray analysis, qPCR	Upregulated expression of AQP4 in the prefrontal cortex of patients with mood disorders.

*AQP4, aquaporin-4; ADCuh, apparent diffusion coefficient from ultra-high b-values; ALDH1L1, aldehyde dehydrogenase 1L1; BA, Brodmann area; BD, bipolar disorder; eDWI, enhanced diffusion-weighted imaging; GFAP, glial fibrillary acidic protein; HC, health control; mRNA, messenger RNA; qPCR, quantitative polymerase chain reaction; SCP, superior cerebellar peduncles*.

However, emerging animal studies provide powerful evidence implying the pathological alterations of astrocytes and AQP4 in depression. Decreased astrocytes and downregulated AQP4 expression have been reported in various animal models of depression (47, 48, 123, 124, 130) (Table 1), supporting dysfunctional glymphatic transport in depression. Effective antidepressant therapy, such as fluoxetine (47, 124, 125), escitalopram (48), mirtazapine (126), ketamine (127, 128), and repetitive high-frequency transcranial magnetic stimulation (TMS) (129) could benefit the functioning of both astrocytes and AQP4, and hence alleviate depressive-like behaviors. Additionally, the synergistic agents of antidepressant—lithium—can attenuate the reduction of AQP4 and disruption of the neurovascular unit in the hippocampus of CUMS rats (130), resulting in a functioning glymphatic system. These therapeutic effects can be suppressed by AQP4 knockout. More specifically, AQP4 deficiency abolishes fluoxetine treatment-induced hippocampal neurogenesis and behavioral improvement in depressive mice (133). Recent studies indicate that the therapeutic option for depression is via the restoration of astrocytes function, AQP4, and glymphatic system (131, 132), which provide further supporting evidence for the critical role of glymphatic flow in depression.

### Abnormalities of Astrocytes and AQP4 in Bipolar Disorders

To date, the role of the glymphatic function in BD has not been widely studied. However, astrocytic dysfunction has undoubtedly been implicated in the development of BD (43). Different from MDD, pictures from human postmortem studies in BD appear to be highly heterogeneous (Table 2). The density of GFAP-positive astrocytes is reported to be significantly increased in Brodmann area (BA) 9 (137) and reduced in BA10 (138), BA24 (38), BA11, and BA 47 (139), while the level of S100β has been reported to be increased in BA40 and reduced in BA9 (140). Other studies on human postmortem tissues from BD exhibit an unchanged density of astrocytes in the frontal cortex (141), cingulate cortex (142), amygdala (41, 143), hippocampus (144), entorhinal cortex (143), basal ganglia (145), dorsal raphe nucleus, and cerebellum (146). The considerable discrepancy is on account of various confounding factors, including phenotype (depressive episode, manic episode, or remission state) (150), cause of death (depressive suicide or physical diseases) (141, 144), comorbidity (150, 151), the methodology used (137, 144), and the brain regions studied (139, 140). Therefore, additional studies regarding diverse phenotypes of BD are essential to investigate state-related abnormalities of astrocytes (152). In patients with bipolar depression, a reduction in S100β-immunopositive astrocytes has been observe, but with no change in GFAP-immunopositive astrocytes (135, 147). As for manic states, *in vivo* studies have revealed increased serum levels of S100β, suggesting astrocytic activation (148).

Upregulated expression of AQP4 in the prefrontal cortex has been revealed in BD (149). Evaluation of the qualitative alterations of astrocytes (especially AQP4 function) is far much valuable than quantitative alterations. The apparent diffusion coefficient from ultra-high b-values (ADC uh), a parameter of enhanced diffusion-weighted imaging (eDWI), can reflect the function of AQP4 (45). In individuals suffering from bipolar depression, increased ADC uh values in bilateral superior cerebellar peduncles (SCP) and cerebellar hemisphere is positively associated with depressive scores, implying that a positive correlation exists between the upregulated expression of AQP4 and severity of depression (46). A plausible explanation is that increased and depolarized AQP4 impair water homeostasis and glymphatic transport in BD (149). Lithium is a classical mood-stabilizer, and its effect of regulating AQP4 function is discussed above (130). Additionally, other mood-stabilizers such as valproic acid, topiramate, and lamotrigine have been shown to inhibit AQP4 (153), and hence regulate directed glymphatic flow.

Even though direct evidence for glymphatic impairment in mood disorders is lacking, astrocytes and AQP4 abnormalities provide support to the hypothesis that glymphatic dysfunction functions as a bridge between sleep disturbance and mood disorders. Additionally, treatments for mood improvement, including medicines, light therapy, sleep invention, and TMS can regulate the function of astrocytes and AQP4. Therefore, AQP4-dependent glymphatic system may serve as a new therapeutic target in mood disorders.

## Conclusion and Outlook

Mood symptoms often occur with the onset of sleep disturbance and ameliorate with improved sleep disturbance. Moreover, early-life sleep problems due to jet-lag, social jet-lag, shift-work, or light pollution can significantly increase the lifetime risk of mood disorders (60). In addition, sleep deprivation can directly trigger mania-like symptoms (80). Based on considerable evidence, a causal relationship between sleep disturbance and mood disorders is hypothesized (154). Therefore, how does disrupted sleep affect the development and phenotype of mood disorders? An intriguing possibility has emerged that glymphatic dysfunction serves as a bridge between sleep disturbance and mood disorders. Adequate sleep, especially deep sleep (NREM III), is a key factor in the functioning of the glymphatic system which accounts for the clearance of metabolic wastes. The effects of sleep on the glymphatic system are mainly dependent on the dynamic alterations of astrocytic function and AQP4 distribution (113, 119, 155). Significantly, suppressed glymphatic circulation, astrocytic abnormalities, and AQP4 depolarization are consistently reported in mood disorders, providing support for the posited hypothesis.

However, several limitations exist in this study. First, much of the existing evidence on the glymphatic system has been conducted in rodents and only a few in humans. Although sleep is an evolutionarily conserved physiological behavior, the reversed circadian rhythms and polyphasic sleep which reduces sleep pressure in rodents make it less representative. Most of the current human studies use invasive methods such as intrathecal injection of contrast agents, while the ADCuh value obtained from the emerging eDWI fails to identify the distribution of AQP4. Therefore, non-invasive methods to explore the glymphatic system in humans are necessary for future studies. Secondly, there is a lack of evidence of known metabolic wastes that fail to be cleared by the glymphatic system and trigger or exacerbate mood symptoms, such as Aβ in AD and α*-synuclein* in PD. Exploring the excessive metabolic wastes in mood disorders is warranted, and can provide promising biomarkers for indicating the occurrence and severity of mood disorders.

## Data Availability Statement

The original contributions presented in the study are included in the article/supplementary material, further inquiries can be directed to the corresponding author/s.

## Author Contributions

TY, YQ, and LY defined the research questions and aims of the study. TY and YQ carried out the literature search, selected and interpreted relevant articles, and wrote the first draft of the manuscript. XY made the original figure and tables. LY and XY critically appraised the texts, figure and tables, corrected them, and made suggestions for further improvement. All authors contributed to the article and approved the submitted version.

## Conflict of Interest

The authors declare that the research was conducted in the absence of any commercial or financial relationships that could be construed as a potential conflict of interest.

## Abbreviations

MDD: major depression disorder
BD: bipolar disorder
AQP4: aquaporin-4
SCN: suprachiasmatic nuclei
SNP: single nucleotide polymorphisms
NREM III: stage 3 non-rapid eye movement sleep
CSF: cerebrospinal fluid
ISF: interstitial fluid
Aβ: amyloid-β
AD: Alzheimer's disease
PD: Parkinson's disease
iNPH: idiopathic normal cranial pressure hydrocephalus
EEG: electroencephalogram
DLMO: dim light melatonin onset
MDR: multifactor-dimensionality reduction
REM: rapid eye movement sleep
MT: melatonin
SSRI: selective serotonin reuptake inhibitor
LHb: lateral habenula
ROS: reactive oxygen species
CNS: central nervous system
PET: positron emission tomography
RBD: REM sleep behavior disorder
DTI: diffusion tensor imaging
ALPS: analysis along the perivascular space
CUMS: chronic unpredictable mild stress
PUFA: polyunsaturated fatty acid
GFAP: glial fibrillary acidic protein
TMS: transcranial magnetic stimulation
BA: Brodmann area
ADC uh: the apparent diffusion coefficient from ultra-high b-values
eDWI: enhanced diffusion-weighted imaging
SCP: superior cerebellar peduncles
PVS: perivascular space
ALDH1L1: aldehyde dehydrogenase 1 family member L1
qPCR: quantitative polymerase chain reaction.
